# Management of Ocular Siderosis: Visual Outcome and Electroretinographic Changes

**DOI:** 10.1155/2016/7272465

**Published:** 2016-03-17

**Authors:** Naresh B. Kannan, Olukorede O. Adenuga, Renu P. Rajan, Kim Ramasamy

**Affiliations:** Aravind Eye Hospitals and Postgraduate Institute of Ophthalmology, 1 Anna Nagar, Madurai, Tamil Nadu 625 020, India

## Abstract

*Purpose.* Ocular siderosis (OS) is a sight threatening complication of retained iron-containing Intraocular Foreign Body (IOFB). Successful localization of the IOFB and timely removal are crucial to its management. The purpose of this study was to review the presentation, management, and outcome of OS at our institution.* Methods*. A retrospective case series of eyes with OS that underwent IOFB removal from January 2009 to March 2015 at our institution.* Results*. OS was seen in 9 eyes of 9 patients during the study period. There were 8 males and 1 female with an age range of 31.6 years. An IOFB was in all the eyes. The most common features of siderosis were cataract and pigmentary retinopathy seen in 6 (67%) and 4 (44%) eyes, respectively. Electroretinogram (ERG) readings were reduced in the 9 eyes. The IOFB was removed by pars plana vitrectomy in all the cases with improvement in ERG amplitudes occurring postoperatively in 7 (78%) eyes.* Conclusion.* A retained iron-containing IOFB can manifest itself after several years with features of OS. A careful clinical and radiologic evaluation is imperative in patients with history suggestive of penetrating ocular injury to rule out retained or occult IOFB and thus prevent this catastrophic condition.

## 1. Introduction

Ocular siderosis (OS) is a severe sequel of retained iron-containing Intraocular Foreign Body (IOFB) [1]. A ferrous IOFB undergoes dissociation resulting in the deposition of iron in the intraocular epithelial structures, notably the lens epithelium, iris and ciliary body epithelium, and the sensory retina, where it exerts a toxic effect on cellular enzyme systems, with resultant cell death [2]. Pigmentary retinopathy followed by atrophy of the retina and Retina Pigment Epithelium (RPE) can have a profound effect on vision and result in a subnormal Electroretinogram (ERG) [2]. Management of OS depends on the successful detection of an occult IOFB as well as the determination of the need and optimum timing for its surgical removal [3, 4]. The aim of this study was to review the presentation, management, and outcome of this relatively rare disorder at our centre.

## 2. Materials and Methods

This was a retrospective study of OS seen at the retina clinic from January 2009 to March 2015. The study was conducted in accordance with the Declaration of Helsinki and ethical approval was obtained from the Institutional Review Board of the hospital. Eyes with a metallic IOFB with clinical and/or ERG features of OS that underwent IOFB removal were included in the study. Exclusion criteria included eyes with clinical features of OS without an ERG, eyes with OS that did not undergo IOFB removal, and eyes with ERG features of OS that had undergone IOFB removal before presentation at our clinic. The cases were identified from the electronic medical records and the case files retrieved. The following information was then extracted from the case files and analysed: demographic data, duration of time from ocular injury to diagnosis of OS, clinical findings, method of IOFB localization, ERG readings at presentation and at 6 months, surgical treatment, complications of treatment, and visual outcome.

All the patients had detailed ocular examination including Visual Acuity (VA) assessment and refraction, slit lamp examination of the anterior segment, dilated indirect fundus examination, and intraocular pressure measurement. Radiological investigations including ocular ultrasonography with or without plain radiographs of the orbit and orbital Computed Tomography (CT) scan were done to localize the IOFB. ERGs done at presentation and posttreatment were analysed. When carrying out the ERG, the patient was first fully dilated and dark adapted for 20 minutes. The patient was then asked to sit with his/her chin on the chin rest of the Ganzfeld bowl with the eye being tested wide open. A contact lens electrode was then placed on the cornea after instilling topical anaesthetic drops. The right eye was tested first with the left occluded. A rod (scotopic) ERG was then recorded with a dim flash of light at −24 decibels. The mixed cone-rod ERG and oscillatory potentials were elicited by a single flash of red light at maximum intensity of 0 decibels. To record the photopic responses from the cone system, the patient underwent 10 minutes of light adaptation by switching on the background light in the Ganzfeld bowl. The cone ERG and 30 Hz flicker ERG were then recorded with a stimulus intensity of 0 decibels.

## 3. Results

**Figure 1:**
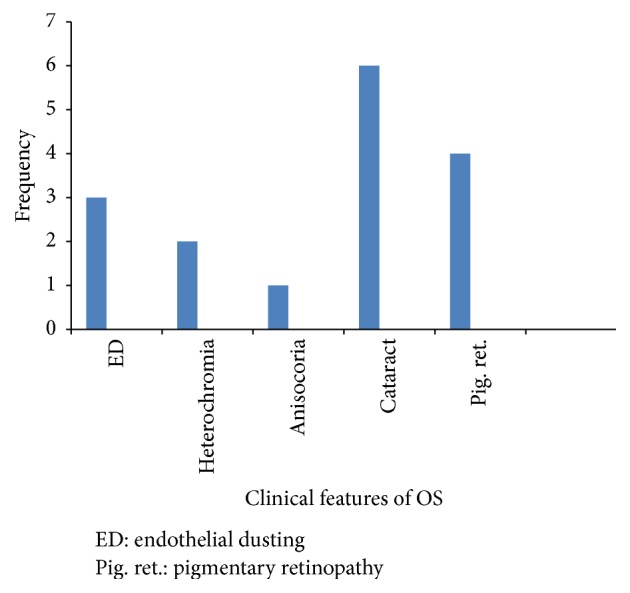
Clinical features of ocular siderosis.

Nine eyes of nine patients with a diagnosis of OS met the inclusion criteria. These were 8 males and 1 female with a mean age of 31.6 years (range: 17–47 years). All the patients gave a history of sustaining ocular injury while hitting a piece of metal on metal. Trauma occurred from 3 months to 12 years (mean: 2.9 years) before the diagnosis of OS was made at our clinic (Table 1). One patient (case 6) had a history of previous intraocular surgery following trauma to the eye but an IOFB was not found then. Best Corrected Visual Acuity (BCVA) at presentation ranged from 1/60 to 6/9 and the most common features of OS were cataract and pigmentary retinopathy seen in 6 (67%) and 4 (44%) eyes, respectively. In four of the cases of cataract, there were iron deposits on the lens capsule suggestive of siderotic cataracts. The other 2 cases had a yellow-brown hue but a histological examination for the presence of iron particles in the lens epithelium was not done. Other features of OS seen were heterochromia, anisocoria, and corneal endothelial dusting (Figure 1). Posterior subcapsular cataract was the most common type of cataract encountered accounting for 5 (83%) of the 6 cases of cataract. Other findings on ocular examination not related to siderosis included corneal scars, iris defects, iridodonesis, dislocated lens, and macular scar. The intraocular pressures were within normal limits in all the patients.

Ocular ultrasonography was done in all the eyes with successful localization of the IOFB in every case. A plain radiograph of the orbit was performed in 4 patients and CT scan in 1 patient with an IOFB demonstrated in each case. All the IOFBs were located in the posterior segment. Surgical treatment included Pars Plana Vitrectomy (PPV) for IOFB removal alone (4 eyes) or in combination with cataract extraction and Intraocular Lens (IOL) implantation (5 eyes) (Table 2). The ERG was subnormal in all the eyes with 7 (78%) eyes showing improvement following surgery (Table 2). Seven (78%) eyes gained 2 or more lines on VA assessment after surgery.

Complications encountered postoperatively included retinal detachment, retinal tears, and cataract progression. Retinal detachment (RD) occurred on 2 occasions in case 4. The first was at 2 months after IOFB removal following which RD surgery was done with anatomical reattachment achieved. Redetachment occurred 5 months later and another surgery was performed with the retina reattached. Multiple horse shoe tears were seen in case 9 and these were successfully barraged with laser. Case 2 had cataract progression and phacoemulsification with IOL implantation was done at 1 year after IOFB removal. Follow-up ranged from 6 months to 3 years.

## 4. Discussion

OS is an uncommon condition and may appear from 18 days up to many years after a penetrating ocular injury with retention of a metallic foreign body [5]. The longest duration between the ocular injury and the diagnosis of OS was 12 years in this series. Sneed and Weingeist [3] and Hope-Ross et al. [6] reported shorter duration of 40 months and 24 months, respectively. The time between ocular trauma and development of OS may be related to the severity of intraocular toxic reactions. This varies depending on the shape and size of the foreign body, its iron content, and the amount of time it remains within the eye [6]. Cataract was the most common feature of OS in this current series. This is similar to the finding by Sneed and colleague [3]. Heterochromia which had a similar incidence to cataract in other studies was, however, only documented in two patients in our series. None of the patients in this present series had secondary glaucoma. The aetiopathogenesis of secondary open-angle glaucoma related to OS has often been ascribed to trabecular fibrosclerosis, probably because of the direct toxic effect of iron ions [7]. Not all cases of metallic IOFB will, however, result in OS. Lim et al. reported a case of an encapsulated iron-containing IOFB situated on the retina of an 84-year-old man for 53 years, which did not lead to the expected OS [8].

Presentation with OS months after an ocular injury may suggest that the IOFB had been missed by the physician or ophthalmologist that attended to the patient following the ocular trauma. All primary care physicians as well as ophthalmologists should be aware of the possibility of a retained IOFB in a penetrating ocular injury particularly when there is a history of high-velocity metallic injury. It should be assumed that ocular injuries sustained in these types of settings potentially harbor an IOFB until proven otherwise [9]. A complete ophthalmic evaluation including imaging studies is therefore essential. The diagnosis of an IOFB is often made by direct visualization on slit lamp examination or ophthalmoscopy. Dilated fundus examination can reveal a foreign body in the vitreous or the retina if the media is not opaque [3]. If the suspected IOFB is not seen, then further evaluation using imaging studies is necessary. Where an IOFB is visualized, confirming the clinical finding with these investigations is also important as the IOFBs may be multiple and may not all be picked up on clinical evaluation.

Imaging studies for the detection of a metallic IOFB include plain radiograph, CT scan, and ocular ultrasonography [4]. Ultrasonography has been shown to be a very valuable tool that can augment the information obtained from other imaging modalities. It is both sensitive and specific for IOFB localization [10]. Farvardin et al. [11] obtained an accuracy of 100% with ultrasonography for IOFB localization. This compares with our finding in this current series. Additional information about the intraocular status can also be obtained simultaneously on ultrasonography. It should therefore be performed in all cases of OS when an IOFB is suspected [4]. Ultrasound biomicroscopy of the anterior segment in eyes with secondary glaucoma may show an anomalous high reflectivity in the deep angular layers. These alterations could be due to metallic particles imprisoned in the trabecular meshwork or indirectly on the consequent fibrotic reaction [12].

Full-field ERG is the most common means for detecting OS and all patients should have this prior to surgical intervention [13]. Iron retinotoxicity leads to a dysfunction of all the layers of the retina with more severe damage occurring in the inner retina than in the outer retina in the late stages of the disease [14]. In the early phase, both the a-wave and the b-wave, though more commonly the former, can be transiently increased. As siderosis progresses, the b-wave decreases, causing the b-wave/a-wave ratio to fall [15]. Rod-dominated responses are predominantly affected as they have a greater susceptibility to iron toxicity compared to the cone system [1]. Eventually, responses are progressively reduced in amplitude to become undetectable [15]. All the patients that had an ERG done in these present series had electrophysiological features of siderosis with improvement in ERG amplitudes following successful removal of the IOFB in 7 patients. Improvement in ERG with removal of an IOFB has been documented by several authors [4, 14, 16, 17]. Contrary to our finding, however, ERG amplitudes remained subnormal in the series by Hope-Ross et al. following IOFB removal [6]. Vision may be excellent in siderosis with ERG amplitudes of up to 50% and complete reversal is possible following successful removal of the IOFB in the early stages of the disease and with amplitudes of up to 40% [4, 14]. Over this limit, macrophagic activity may be overwhelmed by the amount of iron load leading to direct cellular toxicity [13]. Full-field ERG also remains the reference follow-up exam in cases of delayed IOFB removal and embedded IOFB with likely difficulty with surgical removal and also following IOFB removal [13, 15]. Small iron particles can still be released at the inner retinal surface following surgical removal potentially inducing further toxicity [13]. OS with subnormal ERG has been reported 3 years after removal of a metallic IOFB [18].

Removal of the IOFB should be strongly entertained in eyes with diminished ERGs and a mobile foreign body in the vitreous or a nonencapsulated foreign body on the retina [3]. Surgical technique for removal of the retained IOFB is dependent on the site and nature of the IOFB, the clarity of the lens, and whether or not the IOFB is embedded in the retina [5]. The removal may be done via an external approach (sclerotomy with large electromagnet) or an internal approach (vitrectomy followed by forceps or internal magnet use). If the foreign body is located in the posterior vitreous or embedded in the retina, then a PPV is the preferred surgery [7]. Iron-containing foreign bodies may lose their magnetic properties over time and a PPV with removal using intraocular forceps may be necessary [9]. A PPV also has the advantage of providing direct viewing and controlled removal of the IOFB [19]. However, if the lens is clear and the foreign body is not embedded in the retina, then a sclerotomy with magnet extraction or intraocular forceps can be employed to extract the foreign body, particularly if it is located more anteriorly [15]. The presence of a visually significant cataract warrants a combined PPV and cataract extraction [15]. Combined surgery was performed in 5 (56%) patients in this present series. This lies between 25% reported by Hope-Ross and colleagues [6] and 79% reported by Sneed and Weingeist [3].

Visual improvement occurred in 7 (78%) patients in this series following surgery. This is similar to 75% reported by Hope-Ross et al. [6]. The visual potential in eyes with OS may be excellent if the siderotic changes stabilize or improve and if the optic nerve and macula have not been injured [3]. Pollack and Oliver [18] reported an excellent visual outcome in a patient with OS following IOFB removal despite an abnormal ERG. Removal of a cataract at the time of IOFB removal may also lead to significant improvements in vision as was recorded in this series.

This study is limited by its retrospective design. Cases may not have been captured if they were inaccurately diagnosed or coded.

In conclusion, a retained iron-containing IOFB can manifest itself after several years with clinical and electrophysiological features of OS. Removal of the IOFB often leads to improvement in vision and ERG amplitudes. Careful clinical and radiological evaluation is essential following a penetrating ocular injury if this condition is to be prevented.

## Figures and Tables

**Table 1:** Demographic and ocular examination findings.

Case	Age	Sex	Duration between trauma and diagnosis of OS (years)	Cornea	Iris/pupil	Lens	Posterior segment	Location of IOFB
1	26	M	6	Scar	Iris defect	PSC	PR	Inferonasal retina
2	29	M	0.8	ED	Normal	Iron deposits on capsule, PSC	PR	Inferior retina
3	17	M	0.4	Clear	Normal	PSC	PR	Inferior retina
4	32	F	2	ED	Heterochromia	Clear	PR	Inferior retina
5	40	M	0.5	ED	Anisocoria	PSC, iron deposits on capsule		Inferior vitreous
6	36	M	2	Scar	Iris defect	CC, iron deposits on capsule	Vitreous condensation	Inferior retina
7	38	M	0.25	Scar	Iris defect	Clear		Inferior vitreous
8	20	M	2	Normal	Heterochromia	PSC, iron deposits on lens capsule	Vitreous condensation	Anterior vitreous
9	47	M	12	Normal	Iridodonesis	PCIOL		Inferior pars plana

ED: endothelial dusting; PSC: posterior subcapsular cataract; CC: cortical cataract; PCIOL: Posterior Chamber Intraocular Lens; PR: pigmentary retinopathy.

**Table 2:** Treatment, ERG changes, and visual outcome.

Case	Treatment	ERG at presentation	ERG at 6 months	BCVA at presentation	BCVA at last clinic visit
1	PPV + IOFBR	Reduced	Improved	6/60	6/12
2	Phaco. + IOL + PPV + IOFBR	Reduced	Improved	6/18	6/9
3	SICS + PPV + IOFBR	Reduced	Unchanged	6/60	3/60
4	SB + PPV + IOFBR	Reduced	Improved	6/9	6/6
5	Phaco. + IOL + PPV + IOFBR	Reduced	Improved	6/12	6/6
6	Phaco. + IOL + PPV + IOFBR	Reduced	Unchanged	1/60	6/6
7	PPV + IOFBR	Reduced	Improved	6/9	6/6
8	SICS + PPV + IOFBR	Reduced	Improved	5/60	6/18
9	PPV + IOFBR	Reduced	Improved	5/60	6/60

Phaco.: phacoemulsification; SICS: Small Incision Cataract Surgery; SB: Scleral Buckling; IOL: Intraocular Lens; IOFBR: Intraocular Foreign Body Removal; PPV: Pars Plana Vitrectomy.
